# Association between Gout, Urate-Lowering Therapy, and Risk of Developing Type 2 Diabetes Mellitus: A Nationwide Population-Based Retrospective Cohort Study

**DOI:** 10.1155/2020/6358954

**Published:** 2020-07-28

**Authors:** Yi-Jen Fang, Yun-Lung Chung, Cheng-Li Lin, Yun-Ping Lim

**Affiliations:** ^1^Research Center for Environmental Medicine, Kaohsiung Medical University, Kaohsiung, Taiwan; ^2^Ph.D. Program in Environmental and Occupational Medicine, College of Medicine, Kaohsiung Medical University and National Health Research Institutes, Taiwan; ^3^Graduate Institute of Clinical Medicine, Department of Environmental Health, Kaohsiung Medical University, Kaohsiung, Taiwan; ^4^National Institute of Environmental Health Sciences, National Health Research Institutes, Zhunan, Taiwan; ^5^Digestive Disease Center, Show Chwan Memorial Hospital, Changhua, Taiwan; ^6^Research Assistant Center, Show Chwan Health Care System, Changhua, Taiwan; ^7^Department of Medical Research and Development, Chang Bing Show Chwan Memorial Hospital, Changhua, Taiwan; ^8^Management Office for Health Data, China Medical University Hospital, Taichung, Taiwan; ^9^Department of Pharmacy, College of Pharmacy, China Medical University, Taichung, Taiwan; ^10^Department of Internal Medicine, China Medical University Hospital, Taichung, Taiwan; ^11^Department of Medical Research, China Medical University Hospital, Taichung, Taiwan

## Abstract

Gout is the most prevalent inflammatory arthritis in adults. Although the link between gout and type 2 diabetes mellitus (T2DM) has been documented, our understanding of the association between urate-lowering therapy (ULT) among gout patients and T2DM development remains poor. We included 69,326 patients with new-onset gout in 2000-2011. Each case was matched randomly with 1 patient without gout during the study period, and 69,326 patients were recognized as the comparison cohort. A Cox proportional hazard regression model was used to analyze differences in the risk of T2DM development between patients with and without gout after considering related comorbidities. After adjusting for potential confounders, the case group had a higher risk of T2DM than the control cohort (adjusted hazard ratio (aHR) = 1.30, 95%confidence interval (CI) = 1.24-1.38; *P* < 0.001). Gout patients without appropriate ULT had significantly higher risk of T2DM development than the control cohort (aHR = 1.39; 95%CI = 1.30-1.48; *P* < 0.001). Among gout patients, those receiving ULT excluding probenecid (aHR = 0.80; 95%CI = 0.64-1.00), all had significantly lower risk of T2DM than gout patients without ULT (all aHR < 0.90; all *P* < 0.001). In this study, we found that gout increased the risk of T2DM; however, patients with any ULT exhibited a lower risk of T2DM than gout patients without any ULT (all aHR < 0.90, *P* < 0.001; excluding probenecid).

## 1. Introduction

Gout is the most prevalent inflammatory arthritis in adults, and its public health burden is increasing globally. It is characterized by high uric acid (UA) serum levels, which, in turn, could induce the deposition of monosodium urate crystals in the small and large joints [1]. Gout has distinct distribution globally based on prevalence and incidence rates. In the United Kingdom (UK), the prevalence and incidence rates in 2012 were 2.49% and 1.77 per 1,000 person-years, respectively [2]. A high prevalence of gout has also been reported in the USA, at 3.9%, between 2007 and 2008 [3], and in the developed countries, at 0.53–6.1% [4]. The prevalence of gout in Taiwan was 6.24%, with an incidence rate of 2.74 per 1,000 person-years, based on the National Health Insurance Research Database (NHIRD) [5]. Therefore, gout prevalence in the general population is 1 in 16 adults in Taiwan. However, gout management in Taiwan is poor, with only one in five affected people being provided with urate-lowering treatment (ULT).

The global prevalence of type 2 diabetes mellitus (T2DM) after age standardization was estimated to be 9.3% (463 million) in 2019 and is projected to increase to 10.2% (578 million) by 2030 and 10.9% (700 million) by 2045 [6]. The increasing prevalence of gout is associated with several factors including increasing incidence of metabolic syndromes and, in turn, T2DM [7]. Several studies have suggested that gout is an independent risk factor for hypertension, diabetes, insulin resistance, and obesity [7–9]. High UA level is the major indicator of gout and is an independent risk factor for T2DM [10–13]. Guidelines suggest the commencement of ULT when individuals with gout have more than one flare per year [14, 15]. When treating gout with ULT, it is critical that the patient is aware of the risk of depletion of their urate crystal deposits and that it is a lifelong therapy.

The main objective in gout treatment is to reduce or eliminate the pathogenic agents and decrease UA levels to below the saturation point and, in turn, prevent crystallization, while promoting the dissolution of urate crystals. Xanthine oxidase inhibitor (XOI) blocks the synthesis of UA, and an XOI, allopurinol, has been used globally for the treatment of gout patients with renal or cardiovascular disease (CVD) comorbidity, as a first-line medication [16]. Uricosuric drugs, including benzbromarone, sulfinpyrazone, and probenecid, which are second-line gout therapies, block renal tubular urate reabsorption [17]. They act predominantly on urate anion exchanger 1, an organic anion transporter, to prevent the reuptake of UA at the proximal renal tubule, which increases UA renal excretion [17]. Although gout prevalence is high in Taiwan, in addition to the presence of definitive ULT, physicians largely overlook gout during primary care [18]. Furthermore, it is often considered an acute illness rather than a chronic disease, despite its potentially adverse consequences.

We conducted a population-based cohort study using data from the NHIRD in Taiwan to investigate the relationship between gout, ULT use, and the risk of developing T2DM by multivariate retrospective analysis. NHIRD contains comprehensive data on diagnoses, prescriptions, and hospitalizations of practically the entire Taiwan population. We also included numerous comorbidities to disentangle the potential effects of gout on T2DM from the cooccurring effects of these comorbidities.

## 2. Materials and Methods

### 2.1. Data Source

The Taiwan NHIRD was the origin of the data used in the present cohort study. Almost 99% of the health information of the Taiwan population, including demographic data, outpatient, and inpatient visits, and treatment courses are registered in the NHIRD. We analyzed the Longitudinal Health Insurance Database (LHID), which is a subset of the NHIRD, with one million randomly selected subjects. The disease codes used were according to the International Classification of Diseases, 9^th^ Revision, Clinical Modifications (ICD-9-CM). All personal identifiers are removed and encrypted to protect patient privacy. This study was approved by the Institutional Review Board of China Medical University Hospital (CMUH104-REC2-115-CR-4).

### 2.2. Study Population

In the present retrospective cohort study, patients who were newly diagnosed with gout (ICD-9-CM 274) in 2000–2012 were recruited as the case group and those who had never been diagnosed with gout were recruited as the control group. The index date for the case group was the date of gout diagnosis, and that for the control group was assigned randomly. We eliminated subjects aged <18 years of age and who had been diagnosed with T2DM before the index date. For each gout patient, comparisons were randomly selected from the pool of participants without gout and T2DM at the baseline and frequency matched by the year of index date, age, and sex. The observation period was from the index date to the date of T2DM occurrence, date of withdrawal from the NHI program, or December 31, 2013, whichever came first.

### 2.3. Major Outcomes, Comorbidities, and Medications

T2DM (ICD-9-CM 250) was the primary endpoint in the present study. The related comorbidities included hypertension (ICD-9-CM 401-405), stroke (ICD-9-CM 430-438), hyperlipidemia (ICD-9-CM 272), chronic obstructive pulmonary disease (COPD) (ICD-9-CM 491, 492, 496), coronary artery disease (CAD) (ICD-9-CM 410-414), alcohol-related illness (ICD-9-CM 291, 303, 305, 571.0, 571.1, 571.2, 571.3, 790.3, A215, and V11.3), and asthma (ICD-9-CM 493). In the gout patient group, we also considered patients that received different ULTs, such as allopurinol, febuxostat, benzbromarone, sulfinpyrazone, probenecid, and colchicine, which are available in Taiwan.

### 2.4. Statistical Analysis

The differences in the categorical and continuous variables of the case and control groups were analyzed using a chi-squared test and two-sample *t*-test, respectively. We obtained the adjusted hazard ratios (aHRs) and 95% confidence intervals (CIs) using the Cox proportional hazard regression model. The HRs were adjusted for age, sex, and comorbidities in a multivariable regression model. The cumulative incidences of T2DM in the case and control groups were derived using the Kaplan-Meier method, and the differences were examined using the log-rank test. We further analyzed the dose-response effect among patients using antigout treatment. We calculated the average dose of sulfinpyrazone, allopurinol, and colchicine per year by dividing the total prescribed dose by the follow-up period. We classified the patients into two subgroups based on the median. We deemed *P* < 0.05 as indicating statistical significance.

## 3. Results

We identified 69,326 patients aged >18 years with newly diagnosed gout from 2000 to 2012 with at least 2-fold more outpatient or hospitalization visits from principal/secondary diagnoses compared to those of the study group by using the diagnosed date as the index date. We followed the diagnosis guidelines of the American College of Rheumatology criteria, which is the most widely used for the diagnosis of gout [19] as provided in Supplemental Table 1. There were 62,479 (90.1%) outpatients with increased uric acid, and 6,847 (9.88%) inpatients were admitted for gout.

Table 1 presents the demographic, comorbidities, and medication data of the two groups. Participants were mostly male (approximately 70%) and aged less than 49 years (51%). The mean ages of participants with and without gout were 50.4 (±16.4) and 49.8 (±16.6) years, respectively, and the corresponding mean follow-up times were 8.13 (±3.95) and 8.26 (±3.91) years. Patients with gout suffered more comorbidities than gout-free subjects. Most of the participants in the gout group received colchicine (46%) and benzbromarone (44%) medication.

**Figure 1:**
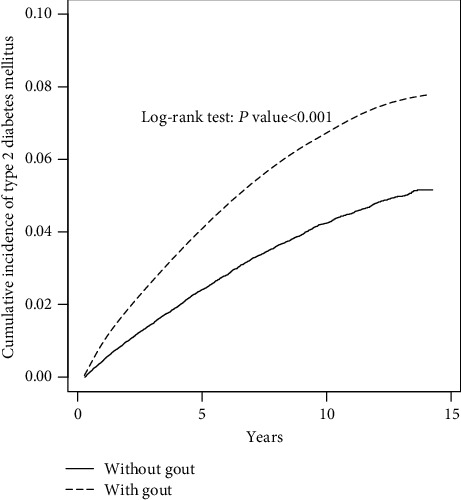
Cummulative incidence of type 2 diabetes mellitus (T2DM) compared between with and without gout using the Kaplan-Meier method. Case group mean follow-up years 8.13 (SD = 3.95). Control group mean follow-up years 8.26 (SD = 3.91).

The Kaplan-Meier analysis (Figure 1) revealed that gout patients had a higher cumulative incidence of T2DM than the controls (*P* < 0.001).  Table 2 presents the results of the stratification analysis. The rate of incidence of T2DM in the control group was 4.42 per 1,000 person-years, and that in the case group was 7.14 per 1,000 person-years. Compared to the case in individuals without gout, the risk of developing T2DM in gout patients was 1.30-fold (*P* < 0.001). In addition, gout increased the risk of T2DM in each stratification (sex, age group, and comorbidity).

The association of ULT and T2DM is demonstrated in Table 3. For patients that received ULT, the aHR of T2DM was 1.39 compared to controls. The risk of developing T2DM in patients treated with sulfinpyrazone (aHR = 0.63; *P* < 0.001) and allopurinol (aHR = 0.83; *P* < 0.001) was relatively low. However, patients who took colchicine increased the risk of T2DM 1.24-fold (*P* < 0.001) relative to the nongout controls. Patients with any ULT exhibited a lower risk of T2DM than gout patients without any ULT (all aHR < 0.90, *P* < 0.001, excluding probenecid).

Compared to that in patients without gout, patients who received sulfinpyrazone treatment at less than 1600 mg per year (aHR = 0.58; 95%CI = 0.41–0.82), patients who received more than 1600 mg of sulfinpyrazone per year (aHR = 0.66; 95%CI = 0.48–0.89), and patients who received less than 1000 mg of allopurinol per year (aHR = 0.73; 95%CI = 0.64–0.84) had a significantly lower risk of T2DM (Table 4). Compared to that in patients without gout, patients who received colchicine treatment for both less than and more than 5 mg per year had a higher risk of T2DM.

## 4. Discussion

In the present study, we used a nationwide population-based cohort study to comprehensively survey the potential association of gout, ULT, and T2DM development and obtained the following key findings: (1) in the gout population, the overall risk of developing T2DM was higher than that in the control population (aHR = 1.30); (2) in the gout population without ULT, T2DM risk was higher in the gout group than in the control group (aHR = 1.39); and (3) with ULT available, the risk of developing T2DM decreased significantly under the febuxostat, sulfinpyrazone, allopurinol, benzbromarone, and colchicine groups.

Out of 23,371,362 participants (men: 49.56%) included within the NHIRD in 2010, 1,458,569 cases of gout were identified, yielding a prevalence of 6.24% (95%CI = 6.23–6.25%) and indicating that about 1 in 16 participants was affected by gout [5]. The prevalence of gout has increased in recent years, reflecting changes in populations due to changes in lifestyles and dietary habits. Despite gout being a widespread chronic disease in Taiwan, only one-third of people with gout are receiving medical management, and only one-fifth are receiving ULT, suggesting that it is managed poorly. Among the patients that received ULT, 60.08% received uricosuric agents exclusively, 28.54% received an XOI alone, while 11.38% received both [5]. In our study, 53.6% received uricosuric agents alone, 21.8% received an XOI alone, and 24.6% received colchicine alone as treatment for gout.

Studies have reported inconsistent findings with regard to the association between gout, ULT, and T2DM. In another study in Taiwan, using a nested case-control study, the authors observed that allopurinol or benzbromarone use was associated with the risk of developing T2DM, particularly in patients reporting prolonged use and high doses [20]. They also reported that ULT may not lower T2DM risk in gout patients younger than 50 years and that ULT could lower T2DM risk in gout patients > 50 years who take low ULT doses. However, they did not compare with the gout patients without treatments. We observed that relatively high rates of gout patients who did not receive ULT were recruited into our study, about 30.7%. Such patients could have relatively high risks of developing T2DM. Gout patients without ULT have a significantly higher (aHR = 1.39) risk of developing T2DM when compared with the nongout population. However, if the gout patients received ULT, their risk of developing T2DM decreased to an aHR of 0.04–0.89. Although our study did not demonstrate that receiving ULTs decreased UA levels, thus, we could not evaluate the influence of the severity of UA in the development of T2DM.

Gout and high levels of UA are associated with increased incidence of several health comorbidities, including CVD [8], chronic kidney disease [21], and T2DM [22, 23]. Since we did not obtain patients' UA laboratory data, we could not determine whether the UA levels following ULT influenced T2DM development. In addition, individuals with gout generally have increased prevalence of several comorbidities that are risk factors for T2DM and could influence T2DM outcome assessments in our study. Consequently, we included the factors and adjusted the HRs for the T2DM risk calculations comprehensively by adjusting for confounders. We observed a more significant increase in T2DM risk in women than in men, with aHRs of 1.40 and 1.26, respectively, which is consistent with the findings of a previous study [24]. In addition, T2DM development risk was inversely correlated with age in the gout patients.

According to a 15-year follow-up study, high UA levels are often associated with the development of hyperinsulinemia, impaired fasting glucose, and T2DM in young adults [25]. The American College of Rheumatology guidelines recommend the maintenance of UA at 6 mg/dL (360 *μ*mol/L) for all patients on ULT [14, 15]. Allopurinol and benzbromarone improve insulin resistance and decrease the risk of developing T2DM [26]. However, most physicians only focus on managing acute attacks rather than long-term therapy so that adherence to ULT is often poor [27].

It was reported that, in 2015-2016, just one-third of gout patients were receiving ULT [28]. In the UK, only 48.48% of the patients were being consulted specifically for gout or treated with ULT, and 37.63% received ULT. In addition, only 18.6% and 27.3% of incident gout patients received ULT within 6 and 12 months of diagnosis, respectively [2]. However, all the estimates are associated with Caucasians, and gout incidence has been hardly estimated in other ethnic groups.

A sustained reduction of UA levels to the target range is critical for the long-term management of gout and to achieve the dissolution of monosodium urate crystals, suppression of acute gout attacks, and resolution of gouty tophi [1]. Reduction of UA levels can be achieved by decreasing monosodium urate crystal production (using XOI such as allopurinol and febuxostat) or increasing UA excretion (using uricosurics, such as benzbromarone, sulfinpyrazone, and probenecid) or metabolism of UA into allantoin, which is more water soluble (using recombinant uricases such as pegloticase and rasburicase), or colchicine (affects the molecular pathology underlying acute and multiple pro- and anti-inflammation associated with gouty arthritis in a multimodal manner). To date, the effects of ULT on blood glucose remain inconclusive. Higher levels of UA have been reported to enter the cell and lower the nitric oxide (NO) levels in the cells, reduce insulin uptake in tissues, reduce glucose transporter 4 translocation, decrease insulin sensitivity, and decrease insulin secretion by pancreatic islet cells and, in turn, increase blood glucose levels [29]. Consequently, the administration of ULT to gout patients would reduce UA levels, in addition to reducing the risk of T2DM development through such mechanisms. Nevertheless, despite increasing evidence from basic research, the accurate mechanisms by which ULT decreases blood glucose remains unclear.

Our study employed a large sample size from a nationwide population-based dataset, reinforcing the statistical power for the assessment of associations between gout and T2DM based on a large and representative population cohort, extracted from the Taiwan NHI system which covers 99% of the population. The patients presented a wide range of demographic characteristics, which facilitated stratified analyses based on age, sex, and comorbidities, without losing precision, while avoiding bias from selection, nonresponse, or poor recall. The LHID has been shown to have appropriate levels of accuracy and completeness based on prescriptions and clinical diagnosis records [30]. In addition, we adjusted for numerous potentially confounding factors that are associated with T2DM over long observation periods. Therefore, the present study showed that gout patients had a higher risk of developing T2DM with a narrow and statistically significant CI.

The present study has limitations that are inherent in any observational study. Admittedly, potential unmeasured confounding variables could still introduce bias into the findings. First, patient data with regard to life habits, smoking status, alcohol consumption, environmental exposure, body mass index, and T2DM family history were unavailable in the LHID. The abovementioned factors may act as confounding factors based on their potential influence of T2DM development. LHID claims data are used mainly for administrative billing purposes. Therefore, the additional information are anonymous. Consequently, it was impossible to contact the patients for additional information directly. Third, no data on dietary effects were available in the present study. Dietary intake could influence the blood glucose levels of a patient. Fourth, there was limited laboratory data in the NHIRD. Therefore, it was not possible to make inferences on underlying relationships among variables, such as UA levels, blood glucose, and glycated hemoglobin. Consequently, we could not determine the UA status in gout patients following ULT. Therefore, our case definition was based on physician-recorded diagnoses instead of the ACR criteria or urate crystal identification.

In our study, gout and diabetes were both diagnosed accurately and coded by specialists according to the standard diagnostic criteria, including typical symptoms and signs, laboratory data, and imaging findings. Therefore, our study minimized the confounding effects of medications by adjusting for comorbidities. However, more information should be obtained from other databases or questionnaires and a prospective study or randomized controlled trial conducted to investigate such a relationship between gout, ULT, and T2DM. Therefore, care of patients with gout could be optimized through physician education, which could promote interest in gout.

## 5. Conclusion

The present study confirms that gout management remains poor, as ULT was prescribed to only 69.3% of gout patients, while 30.7% of gout patients did not receive ULT, potentially leading to the elevated UA levels and increased gout flares, with major adverse consequences such as T2DM. However, patients with any ULT exhibited a lower risk of T2DM than gout patients without any ULT. Future pharmacoepidemiological studies should be conducted to investigate the specific putative link between ULT and T2DM risk among patients with gout. Additionally, whether a dose threshold or dose-gradient effects of ULT exist in the risk of T2DM development should be investigated in future. Our results provide insights that could facilitate the development of recommendations and guidelines for the clinical management of gout patients and the effective mitigation of any long-term consequences such as T2DM development.

## Figures and Tables

**Table 1:** Demographic characteristics, comorbidity, and medication in patients with and without gout.

Variable	Gout		*P* value
	No	Yes	
	*N* = 69326	*N* = 69326	
Sex	*n* (%)	*n* (%)	0.37
Female	20575 (29.7)	20422 (29.5)	
Male	48751 (70.3)	48904 (70.5)	
Age, mean (SD)	49.8 (16.6)	50.4 (16.4)	<0.001
Stratify age			0.99
≤49	35211 (50.8)	35211 (50.8)	
50-64	19471 (28.1)	19471 (28.1)	
>65	14644 (21.1)	14644 (21.1)	
Comorbidity			
Hypertension	14556 (21.0)	27435 (39.6)	<0.001
Stroke	1637 (2.36)	2031 (2.93)	<0.001
Hyperlipidemia	7084 (10.2)	22309 (32.2)	<0.001
COPD	5472 (7.89)	7828 (11.3)	<0.001
CAD	6791 (9.80)	11933 (17.2)	<0.001
Alcohol-related illness	1654 (2.39)	3362 (4.85)	<0.001
Asthma	3199 (4.61)	5078 (7.32)	<0.001
Medication			
Allopurinol		11245 (16.2)	
Febuxostat		248 (0.36)	
Benzbromarone		30368 (43.8)	
Sulfinpyrazone		2363 (3.41)	
Probenecid		358 (0.52)	
Colchicine		31975 (46.1)	

Chi-squared test.

**Table 2:** Comparison of incidence and hazard ratio (HR) of type 2 diabetes mellitus (T2DM) stratified by sex, age, and comorbidity between with and without gout.

Variable	Gout							
	No			Yes				
	Event	PY	Rate^#^	Event	PY	Rate^#^	Crude HR (95% CI)	Adjusted HR^†^ (95% CI)
All	2,530	572,908	4.42	4,023	563,653	7.14	1.61 (1.53, 1.69)^∗∗∗^	1.30 (1.24, 1.38)^∗∗∗^
Sex								
Female	832	165,876	5.02	1,460	162,322	8.99	1.79 (1.64, 1.95)^∗∗∗^	1.40 (1.28, 1.53)^∗∗∗^
Male	1,698	407,032	4.17	2,563	401,331	6.39	1.53 (1.44, 1.63)^∗∗∗^	1.26 (1.18, 1.34)^∗∗∗^
Stratify age								
≤49	551	310,196	1.78	1,252	308,586	4.06	2.28 (2.07, 2.52)^∗∗∗^	1.66 (1.49, 1.84)^∗∗∗^
50-64	1,022	156,875	6.51	1,492	154,273	9.67	1.48 (1.37, 1.60)^∗∗∗^	1.18 (1.08, 1.29)^∗∗∗^
>65	957	105,836	9.04	1,279	100,794	12.7	1.40 (1.28, 1.51)^∗∗∗^	1.21 (1.11, 1.32)^∗∗∗^
Comorbidity^‡^								
No	1054	404549	2.61	847	243410	3.48	1.34 (1.22, 1.46)^∗∗∗^	1.58 (1.44, 1.73)^∗∗∗^
Yes	1476	168359	8.77	3176	320244	9.92	1.15 (1.08, 1.22)^∗∗∗^	1.25 (1.17, 1.33)^∗∗∗^

^#^Rate: incidence rate, per 1,000 person-years; crude HR: crude hazard ratio. ^†^Adjusted HR: multivariable analysis including age, sex, and comorbidities of hypertension, stroke, hyperlipidemia, COPD, CAD, alcohol-related illness, and asthma. ^‡^Comorbidity: patients with any one of the comorbidities (hypertension, stroke, hyperlipidemia, COPD, CAD, alcohol-related illness, and asthma) were classified as the comorbidity group. ^∗^*P* < 0.05; ^∗∗^*P* < 0.01; ^∗∗∗^*P* < 0.001.

**Table 3:** Incidence, crude, and adjusted hazard ratio (aHR) of type 2 diabetes mellitus (T2DM) compared among gout patients with and without antigout treatment compared to nongout controls.

Variables	*N*	Event	PY	Rate^#^	Crude HR (95% CI)	Adjusted HR^†^ (95% CI)	Adjusted HR^†^ (95% CI)
Nongout controls	69,326	2,530	572,908	4.42	1 (reference)	1 (reference)	
Gout without the following antigout treatment	21,291	1,556	159,672	9.74	2.17 (2.04, 2.31)^∗∗∗^	1.39 (1.30, 1.48)^∗∗∗^	1 (reference)
Gout with antigout treatment							
Febuxostat	248	0	2,475	0.00	—	—	0.04 (0.01, 0.17)^∗∗∗^
Probenecid	357	22	3,648	6.03	1.45 (0.95, 2.20)	0.92 (0.61, 1.41)	0.80 (0.64, 1.00)
Sulfinpyrazone	2,281	74	20,757	3.57	0.82 (0.65, 1.04)	0.63 (0.50, 0.79)^∗∗∗^	0.57 (0.51, 0.64)^∗∗∗^
Allopurinol	10,247	432	94,179	4.59	1.07 (0.96, 1.18)	0.83 (0.75, 0.92)^∗∗∗^	0.57 (0.54, 0.61)^∗∗∗^
Benzbromarone	23,109	1,306	193,304	6.76	1.53 (1.43, 1.64)^∗∗∗^	1.05 (0.98, 1.12)	0.89 (0.86, 0.93)^∗∗∗^
Colchicine	11,793	633	89,618	7.06	1.57 (1.43, 1.71)^∗∗∗^	1.24 (1.13, 1.35)^∗∗∗^	0.72 (0.68, 0.76)^∗∗∗^

^#^Rate: incidence rate, per 1,000 person-years; crude HR: crude hazard ratio. ^†^Adjusted HR: multivariable analysis including age, sex, and comorbidities of hypertension, stroke, hyperlipidemia, COPD, CAD, alcohol-related illness, and asthma. ^∗^*P* < 0.05; ^∗∗^*P* < 0.01; ^∗∗∗^*P* < 0.001.

**Table 4:** Incidence and adjusted hazard ratios of type 2 diabetes mellitus (T2DM) stratified by average days used per year, average dose per year, and average days per year of antigout therapy.

Medication exposed	*N*	Event	Person-years	Rate	aHR (95% CI)^a^
Sulfinpyrazone^#^					
Nongout control	69,326	2,530	572,908	4.42	1.00
≤1600 mg	1,204	34	11,783	2.89	0.58 (0.41, 0.82)^∗∗^
>1600 mg	1,229	43	10,206	4.21	0.66 (0.48, 0.89)^∗∗^
Allopurinol^#^					
Nongout control	69,326	2,530	572,908	4.42	1.00
≤1000 mg	6,021	217	60,065	3.61	0.73 (0.64, 0.84)^∗∗^
>1000 mg	6,139	303	52,346	5.79	0.90 (0.80, 1.02)
Colchicine^#^					
Nongout control	69,326	2,530	572,908	4.42	1.00
≤5 mg	4,769	324	35,983	9.00	1.63 (1.45, 1.83)^∗∗∗^
>5 mg	37,207	1,863	319,385	5.83	1.14 (1.07, 1.22)^∗∗∗^

^#^Average dose used per year is partitioned into two segments by median. ^#^Rate: incidence rate, per 1,000 person-years; crude HR: crude hazard ratio. ^†^Adjusted HR: multivariable analysis including age, sex, and comorbidities of hypertension, stroke, hyperlipidemia, COPD, CAD, alcohol-related illness, and asthma. ^∗^*P* < 0.05; ^∗∗^*P* < 0.01; ^∗∗∗^*P* < 0.001.

## Data Availability

The data used to support the findings of this study are included within the article.

## Supplementary Materials

Supplementary Materials Supplemental Table 1: 1977 American College of Rheumatology criteria for diagnosis of gout (adapted from [19]).
