# Supplementary material for: Association between Gout, Urate-Lowering Therapy, and Risk of Developing Type 2 Diabetes Mellitus: A Nationwide Population-Based Retrospective Cohort Study
Source: Biomed Res Int. 2020 Jul 28;2020:6358954. doi: 10.1155/2020/6358954 (PMC7407011; doi:10.1155/2020/6358954)
Supplement: Supplementary Materials — Supplemental Table 1: 1977 American College of Rheumatology criteria for diagnosis of gout (adapted from [19]). [file 6358954.f1.doc]

**Supplemental Table 1.** 1977 The American College of Rheu­matology criteria for diagnosis of gout (adapted from [19]).

| A. Presence of characteristic urate crystals in the joint fluid |
| --- |
| B. Presence of a tophus proven to contain urate crystals by chemical means or polarized light microscopy |
| C. Presence of 6 or more of the following clinical, laboratory, or radiologic findings: |
| (1) More than one attack of acute arthritis |
| (2) Development of maximal inflammation within one day |
| (3) Attack of monoarticular arthritis |
| (4) Joint redness |
| (5) Pain or redness in the first metatarsophalangeal joint |
| (6) Unilateral attack involving first metatarsophalangeal joint |
| (7) Unilateral attack involving tarsal joint |
| (8) Suspected tophus |
| (9) Hyperuricemia |
| (10) Asymmetric swelling within a joint on radiography |
| (11) Subcortical cyst without erosions on radiography |
| (12) Culture of joint fluid negative for microorganisms during attack of joint inflammation |
